# Supplementary material for: Regulation of VEGFR2 and AKT Signaling by Musashi-2 in Lung Cancer
Source: Cancers (Basel). 2023 Apr 28;15(9):2529. doi: 10.3390/cancers15092529 (PMC10177017; doi:10.3390/cancers15092529)
Supplement: Supplementary file 1 [file cancers-15-02529-s001.zip › Fig_legends.pdf]

Figure 1.

MSI2 directly binds VEGFR2 mRNA in human NSCLC. **(A)** Western blot of human NSCLC cell lines. **(B)** RT-qPCR analysis of VEGFR2 mRNA from human NSCLC cell lines. Data from at least three independent experiments by Image J software and normalized to 18S rRNA and to A549. **(C)** MSI2 consensus binding sites in human mRNAs. Location of consensus binding sites for Musashi proteins in the noted human genes, as defined from studies by Bennett et al[1], and Wang et al[2]. Coding sequences are represented by thick lines; 3' untranslated regions by a thin line. 7- or 8-bp consensus sequences are indicated by arrows. VEGFR2 reference sequence - NCBI Reference Sequence: NM_002253.4, HIF1 reference sequence - NCBI Reference Sequence: NM_001243084.2. **(D)** Quantification of mRNA immunoprecipitation (RIP) results from assays performed in Hcc1171 and H441 cell lysates using antibodies to MSI2, or IgG (negative control) antibodies, followed by quantitative RT-PCR. Data are normalized to positive control *PTP4A1, TGFBR1*, and *SMAD3* are additional positive controls; *GAPDH* and *ACTB* are negative control. The data shown reflect the average of three independent RIP experiments. Error bars represented by SEM. Statistical analysis was performed using an unpaired two-tailed t-test. *p < 0.05, **p < 0.01, ***p < 0.001 for all graphs.

Figure 2.

MSI2 regulation of VEGFR2 and VEGF-A protein levels in human NSCLC cell lines. **(A)** Western blots of indicated cell lines, following depletion by shRNA (sh1, sh2) and siRNA (si1, si2) of MSI2. Negative controls include pLKO and SCR. MSI2 depletion was induced by the addition of 1 µg/ml of Doxycycline for 48 h. **(B)** Quantification of Western blot (Fig 2A) data from at least three independent experiments by Image J software, with values normalized to negative control and β-actin. **(C)** The concentration of VEGF-A in cell culture media from indicated cell lines, following depletion by shRNA (sh1, sh2), siRNA (si1, si2) and overexpression (MSI2) of MSI2. MSI2 depletion was induced by the addition of 1 µg/ml of Doxycycline for 48 h. The ELISA data shown reflects the average of three independent experiments. Error bars represented by SEM. Statistical analysis was performed using an unpaired two-tailed t-test. *p < 0.05, **p < 0.01, ***p < 0.001 for all graphs.

Figure 3.

VEGFR2-independent effects of MSI2 KD on AKT signaling. (A) Western blots of indicated cell lines, following depletion by shRNA (sh1, sh2) and siRNA (si1, si2) of MSI2. Negative controls include pLKO and SCR. **(B)** Quantification of Western blot data (Fig. 3A) from at least three independent experiments by Image J software, with values normalized to negative control and β-actin. **(C)** Rescue experiment: western blots of A549 and H441 cell lines, following depletion by shRNA (sh1) of MSI2 and VEGFR2 overexpression (VEGFR2 OE). Negative control is pHAGE. **(D)** Quantification of Western blot data (Fig. 3C) from at least three independent experiments by Image J software, with values normalized to empty vector and β-actin. **(E)** Western blot of indicated cell lines, following depletion by shRNA (sh1) of MSI2 and VEGFR2 overexpression (VEGFR2 OE). **(F)** Quantification of Western blot data (Fig. 3E) from at least three independent experiments by Image J software, with values normalized to empty vector and β-actin. Error bars represented by SEM. Statistical analysis was performed using an unpaired two-tailed t-test. *p < 0.05, **p < 0.01, ***p < 0.001, ****p <0.0001.

Figure 4.

Expression of MSI2, VEGFR2 and VEGF-A proteins in human NSCLC patient samples. (A) Representative IHC images of MSI2 and VEGFR2 expression in normal lung and lung tumors. **(B)** MSI2 and VEGF-A (n=94), MSI2 and VEGFR2 (n=118) H-score correlation of human NSCLC TMAs. For IHC quantification, each spot was examined by board- certified pathologists (ED and NK) who assigned a score of 0 (no staining), 1+ (weak staining), 2+ (moderate staining), and 3+ (strong staining) within carcinomatous areas. The score for each of the two tumor spots was averaged for statistical analysis. The H-score, which ranges from 0 to 300, was calculated using the following formula: [1x(% cells 1+) + 2x(% cells 2+) + 3x(% cells 3+)], which reflects staining intensity as well as percentage of positive cells. A sum of p2 and p3 represents a sum of 2+ and 3+ cells (2x (% cells 2+) + 3x(% cells 3+), which excludes 1+ cells.

Supplementary Figure S1.

MSI2 regulation of VEGFR2 protein level in a mouse cell line. **(A)** Heatmap of RPPA results for VEGFR2 protein expression in 344SQ cell line transfected with empty vector or shRNAs or MSI2 depletion with two independent shRNAs (M2-m1 and M2-m2). **(B)** Western blots of 344SQ cell line lysates, following depletion (MSI2 KD sh1, sh2) and overexpression (MSI2 OE, MSI2) of MSI2. pLKO and pLV are negative controls. MSI2 depletion was induced by the addition of 1 μg/ml of Doxycycline for 48 h. **(C)** Quantification of Western blot data (Supplementary Fig. 1A) from at least three independent experiments by Image J software, with values normalized to negative control and β-actin. **(D)** RT-qPCR analysis of Vegfr2 and Vegf-a mRNA from 344SQ cell line following depletion (MSI2 KD, sh1, sh2) and overexpression (MSI2 OE, MSI2) of MSI2. pLKO and pLV are negative controls. MSI2 depletion was induced by the addition of 1 μg/ml of Doxycycline for 48 h. Data normalized to negative control and Polr2a. Error bars represented by SEM. Statistical analysis was performed using an unpaired two-tailed t-test. *p < 0.05, **p < 0.01, ***p < 0.001 for all graphs.

Supplementary Figure S2. (A) Quantification of mRNA immunoprecipitation (RIP) results from assays performed in A549 cell lysates using antibodies to MSI2, or IgG (negative control) antibodies, followed by quantitative RT-PCR. Data are normalized to positive control *PTP4A1*, *TGFBR1*, and *SMAD3* are additional positive controls; *GAPDH* and *ACTB* are a negative control. The data shown reflect the average of three independent RIP experiments. **(B)** Western blots of indicated cell lines, following depletion by shRNA (sh1, sh2) and siRNA (si1, si2) of MSI2. Negative controls include pLKO and SCR. **(C)** Western blot of human NSCLC cell lines, following MSI2 overexpression (MSI2). Negative control is empty vector pLV. **(D)** Quantification of Western blot data (Supplementary Fig. 2C) from at least three independent experiments by Image J software, with values normalized to negative control and β-actin. Error bars represented by SEM. Statistical analysis was performed using an unpaired two-tailed t-test. *p < 0.05, **p < 0.01, ***p < 0.001.

Supplementary Figure S3. Consequences of MSI2 depletion (left) and overexpression (right) on mRNA expression levels of *VEGFR2* and *VEGF-A*. Quantitative RT-PCR of mRNA of human NSCLC cell lines, following MSI2 depletion by shRNA (sh1, sh2) and siRNA (si1, si2) and overexpression (MSI2) of MSI2. Negative controls include pLKO, SCR, pLV. Error bars

represented by SEM. Statistical analysis was performed using an unpaired two-tailed t-test. *p < 0.05, **p < 0.01, ***p < 0.001.

Supplementary Figure S4. (A) Cell viability quantified by Cell Titer Blue (CTB) assay of indicated cell lines following depletion by shRNA (sh1, sh2), siRNA (si1, si2) and overexpression (MSI2) of MSI2. MSI2 depletion was induced by the addition of 1 µg/ml of Doxycycline for 48h. Negative controls include pLKO or SCR. Error bars represented by SEM. Statistical analysis was performed using an unpaired two-tailed t-test. *p < 0.05, **p < 0.01, ***p < 0.001, ****p <0.0001. **(B)** Western blots of indicated cell lines, following depletion by shRNA (sh1, sh2) and siRNA (si1, si2) of MSI2. Negative controls include pLKO and SCR. Error bars represented by SEM. Statistical analysis was performed using an ANOVA. *p < 0.05, **p < 0.01, ***p < 0.001, ****p <0.0001.
