# Supplementary material for: Regulation of VEGFR2 and AKT Signaling by Musashi-2 in Lung Cancer
Source: Cancers (Basel). 2023 Apr 28;15(9):2529. doi: 10.3390/cancers15092529 (PMC10177017; doi:10.3390/cancers15092529)
Supplement: Supplementary file 1 [file cancers-15-02529-s001.zip › Supp Table S2.pdf]

Name	Vector	Type of insert	Type of expression	Cell line origin
A549 pLKO	Tet-pLKO-puro	Empty	No	Human
A549 sh1	Tet-pLKO-sh1-puro	MSI2-shRNA1	Inducible	Human
A549 sh2	Tet-pLKO-sh2-puro	MSI2-shRNA2	Inducible	Human
A549 pLV	pLV-CMV-puro	Empty	No	Human
A549 MSI2	pLV-CMV-MSI2-puro	MSI2 ORF	Constitutive	Human
A549 pHAGE	Tet-pLKO-puro<br>pHage-hygro	Empty<br>Empty	No<br>No	Human
A549 pHAGE MSI2 sh1	Tet-pLKO-sh1-puro<br>pHage-hygro	MSI2-shRNA1<br>Empty	Inducible<br>No	Human
A549 VEGFR2 OE	Tet-pLKO-puro<br>pHage-KDR-hygro	Empty<br>KDR ORF	No<br>Constitutive	Human
A549 VEGFR2 OE MSI2 sh1	Tet-pLKO-sh1-puro<br>pHage-KDR-hygro	MSI2-shRNA1<br>KDR ORF	Inducible<br>Constitutive	Human
H441 pLKO	Tet-pLKO-puro	Empty	No	Human
H441 sh1	Tet-pLKO-sh1-puro	MSI2-shRNA1	Inducible	Human
H441 sh2	Tet-pLKO-sh2-puro	MSI2-shRNA2	Inducible	Human
H441 pLV	pLV-CMV-puro	Empty	No	Human
H441 MSI2	pLV-CMV-MSI2-puro	MSI2 ORF	Constitutive	Human
H441 pHAGE	Tet-pLKO-puro<br>pHage-hygro	Empty<br>Empty	No<br>No	Human
H441 pHAGE MSI2 sh1	Tet-pLKO-sh1-puro<br>pHage-hygro	MSI2-shRNA1<br>Empty	Inducible<br>No	Human
H441 VEGFR2 OE	Tet-pLKO-puro<br>pHage-KDR-hygro	Empty<br>KDR ORF	No<br>Constitutive	Human
H441 VEGFR2 OE MSI2 sh1	Tet-pLKO-sh1-puro<br>pHage-KDR-hygro	MSI2-shRNA1<br>KDR ORF	Inducible<br>Constitutive	Human
Calu-1 pLKO	Tet-pLKO-puro	Empty	No	Human
Calu-1 sh1	Tet-pLKO-sh1-puro	MSI2-shRNA1	Inducible	Human
Calu-1 sh2	Tet-pLKO-sh2-puro	MSI2-shRNA2	Inducible	Human
Calu-1 pLV	pLV-CMV-puro	Empty	No	Human
Calu-1 MSI2	pLV-CMV-MSI2-puro	MSI2 ORF	Constitutive	Human
Hcc1171 pLV	pLV-CMV-puro	Empty	No	Human
Hcc1171 MSI2	pLV-CMV-MSI2-puro	MSI2 ORF	Constitutive	Human
Hcc1171 pHAGE	pHage-hygro	Empty	No	Human
Hcc1171 VEGFR2 OE	pHage-KDR-hygro	KDR ORF	Constitutive	Human
344SQ pLKO	pLKO.1-puro	Empty	No	Mouse
344SQ sh1	pLKO-m1-puro	MSI2-shRNA1	Constitutive	Mouse
344SQ sh2	pLKO-m2-puro	MSI2-shRNA2	Constitutive	Mouse

Supplementary table S2. List of cell line derivatives used in the study. For human cell lines, the lentiviral vector Tet-pLKO-puro (Addgene, Plasmid #21915) was used for the inducible expression of shRNAs. For murine cell lines, the lentiviral vectors pLKO.1-puro (Addgene, Plasmid #8453) was used for the stable expression of shRNAs. For human cell lines, the pLV-

CMV-puro vector (a kind gift from Dr. A. Ivanov, West Virginia University) was used for the stable expression of the MSI2 cDNA, the pHage-KDR-hygro vector was used for the stable expression of the VEGFR2 cDNA.
