# Supplementary material for: Regulation of VEGFR2 and AKT Signaling by Musashi-2 in Lung Cancer
Source: Cancers (Basel). 2023 Apr 28;15(9):2529. doi: 10.3390/cancers15092529 (PMC10177017; doi:10.3390/cancers15092529)
Supplement: Supplementary file 1 [file cancers-15-02529-s001.zip › Supp Table S3.pdf]

siRNA Catalog Number	Gene Symbol	Sequence
SI04236652 and SI04285834	Human MSI2 "-si1" (Mixture 2)	ATGAGAGATCCCACTACGAAA and CUGGAUUGGUCAUCAGAUU
SI04312665 and SI04375847	Human MSI2 "-si2" (Mixture 1)	TCCCAACTTCGTGGCGACCTA and CCAGATAGCCTTAGAGACTAT

Supplementary table S3. siRNAs targeting human and murine MSI2. siRNA reference numbers are from Qiagen (Frederick, MD).
