# Supplementary material for: Regulation of VEGFR2 and AKT Signaling by Musashi-2 in Lung Cancer
Source: Cancers (Basel). 2023 Apr 28;15(9):2529. doi: 10.3390/cancers15092529 (PMC10177017; doi:10.3390/cancers15092529)
Supplement: Supplementary file 1 [file cancers-15-02529-s001.zip › Supp Table S4.pdf]

Gene symbol (H)-human; (M)-mouse	SYBR Green	Taqman Life Technologies
PTP4A1 (H)	Fw: 5`-ATCCAACCAATGCGACCTTA<br>Rev: 5`-AAGGCCAATCAAGAACATGG	
GAPDH (H)	Fw: 5`-TGCACCACCAACTGCTTAGC<br>Rev: 5`-GGCATGGACTGTGGTCATGAG	
TGFBR1 (H)	Fw: 5`- ATCTTGTACCTTCTGACCCATC<br>Rev: 5`- TGGCATACCAACATTCTCTCAT	
VEGFR2 (H)	Fw: 5`-CAGAAATGTACCAGACCATGCT<br>Rev: 5`- GGAAGAACAATGTAGTCTTTGCC	
VEGF-A (H)	Fw: 5`- CCATGAACTTTCTGCTGTCTTG<br>Rev: 5`- GCGCTGATAGACATCCATGA	
ERBB3 (H)	Fw: 5`-TGCTATACAGTGAGGCCAAGACTC<br>Rev: 5`-CAACTCCCAAACTGTCACACCATA	
SMAD3 (H)	Fw: 5`-CCAGCACATAATAACTTGGACCT<br>Rev: 5`-GATGTGTCTCCGTGTCAGCTC	
MSI2 (H)	Fw: 5`-GGTCATGAGAGATCCCACTACG<br>Rev: 5`-TCTACACTTGCTGGGTCTGC	
ACTB (H)	Fw: 5`- TTGTTACAGGAAGTCCCTTGCC<br>Rev: 5`- ATGCTATCACCTCCCCTGTGTG	
PTEN (H)	Fw: 5`- GCTCTATACTGCAAATGCTATCG<br>Rev: 5`- CCACAAACAGAACAAGATGCT	
18S rRNA (H)		Fw: 5` GCTCTTTCTCGATTCCGT<br>Rev: 5`- CCAGAGTCTCGTTCGTTATC<br>Probe: 6fam-TTCTTAGTTGGTGGAGCGATTTGT-Iowa blackFQ
Msi2 (M)	Fw: 5`-AGCAGTATTTCGAGCAGTTTGGCA<br>Rev: 5`-TGTCGAACATCAGCATCGCATCC	
Vegfr2 (M)	Fw: 5`-TTCAGAGTTGGTGGAGCATT<br>Rev: 5`-CTCTTCCATGCTCAGTGTCTC	
Vegfa (M)	Fw: 5`-AGAAAGACAGAACAAAGCCAGA<br>Rev: 5`-TGGTGACATGGTTAATCGGT	
Polr2a(M)	Fw: 5`- GGTCCTTCGAATCCGCATC<br>Rev: 5`- CAGGGTCATATCTGTCAGCATG	

Supplementary Table S4. **Primers used for RT-PCR to quantify gene expression.** List of primers used for SYBR Green assays, and TaqMan gene expression assays, used for the qRT-PCR analysis of gene expression and RNA immunoprecipitation.
