# Supplementary material for: Regulation of VEGFR2 and AKT Signaling by Musashi-2 in Lung Cancer
Source: Cancers (Basel). 2023 Apr 28;15(9):2529. doi: 10.3390/cancers15092529 (PMC10177017; doi:10.3390/cancers15092529)
Supplement: Supplementary file 1 [file cancers-15-02529-s001.zip › Supp Table S8_adeno.pdf]

Gender	%
Male	47.4
Female	52.6
**Age at diagnosis**	**Years**
Mean	62.9
Min	38
Max	81
SD	10.7
**Histology**	**%**
Squamous	0
Non-squamous	100
**Overall stage**	**%**
1	12
2	8.6
3	57.9
4	15.5
**T stage**	**%**
0	0.9
1	15.5
2	57.7
3	8.7
4	16.3
X	0.9
**Lymph Nodes**	**%**
0	33.6
1	12
2	47.4
3	4.4
x	2.6
**M stage**	**%**
0	81.9
1	15.5
x	2.6

Supplementary Table S8, Clinical characteristics of NSCLC specimens from Rostov National Research Cancer Institute Human Tissue Repository Facility (Rostov-on-Don, Russia), Republican Clinical Oncology Dispensary named after Prof. M.Z. Sigal (Kazan, Russia) and Fox Chase Cancer Center (Philadelphia, USA). IHC contained specimens from 116 patients with characteristics noted in the table.
